# Paraspinal and Extensive Epidural Abscess: The Great Masqueraders of Abdominal Pain

**DOI:** 10.1155/2015/103624

**Published:** 2015-12-06

**Authors:** Andrew Chu, Thu Thu Aung, Uday Shankar

**Affiliations:** Aventura Hospital and Medical Center, Internal Medicine Residency Department, 20900 Biscayne Boulevard, Aventura, FL 33180, USA

## Abstract

Paraspinal and epidural abscesses are rare conditions often diagnosed later in the disease process that can have significant morbidity and mortality. Predisposing risk factors include diabetes, human immunodeficiency virus, intravenous drug abuse, and previous history of spinal surgery or injection. They can threaten the spinal cord by compressive effect, leading to sensory motor deficits and ultimately paralysis and death. Diagnosis may be a challenge due to the delayed presentation of nonspecific back pain or radicular pain such as chest pain or abdominal pain. We present a rare case on a patient with periumbilical pain, constipation, and urinary retention who was ultimately diagnosed with a paraspinal abscess extending into the epidural space from T1 to S2. He underwent decompressive laminectomy with incision and drainage of the abscesses. The patient made an excellent recovery postoperatively, and repeat magnetic resonance imaging at six weeks showed resolution of the abscess.

## 1. Introduction

Paraspinal and epidural abscesses are rare medical emergencies, estimated to be about 0.2–1.2 cases per 10,000 hospital admissions [1]. The meta-analysis found the predisposing risk factors to the development of epidural abscess include diabetes (15–53.7% cases), spinal surgery (22%), and intravenous drug use (8.8%). A few of the cases are reported to be related to alcoholism and trauma [2]. In approximately one-third of the cases, a source of infection cannot be identified [3]. Clinical outcome can be catastrophic if left untreated.

In a meta-analysis of 915 patients diagnosed with epidural abscesses, the common presenting symptoms were back pain (71%), fever (66%), and paralysis (34%) in later stages [4]. Some symptoms include headache, bladder and bowel dysfunction, irritability, and abdominal pain [5]. There have been only a few published case reports in medical literature regarding abdominal pain as the main presenting feature of epidural abscesses [2, 5–8]. Even less literature has been published on the topic of paraspinal abscesses. To our knowledge, there has not been a case of paraspinal and extensive epidural abscess presenting with a vague abdominal pain. We present a rare case of a young man who exhibited acute onset of periumbilical pain, constipation, and urinary retention. A paraspinal abscess extending into epidural space from T1 to S2 was subsequently found. This case serves to emphasize a high index of clinical suspicion for paraspinal and epidural abscess in someone who is presenting with vague abdominal pain in the absence of intra-abdominal pathology.

## 2. Case Presentation

A 25-year-old Chinese American male came to the emergency department with two days of abdominal pain. The pain was sharp, localized around periumbilical, and radiated toward bilateral flank in a band-like formation. Pain was at the worst during supine and improved in erect body position. His pain was not associated with lateral flexion of his hip, flexion, or extension of his lumbar spine. He also complained of subjective fever, constipation, and urinary retention. He denied having chills, nausea, emesis, anorexia, diarrhea, chest pain, shortness of breath, trauma, weakness, numbness, or trouble walking. He was prescribed senna, tramadol, and tamsulosin for his symptoms a day prior to an urgent care visit. He did not have prior sick contact. No past medical or surgical history was reported. Family history was only positive for hypertension in father. He denied smoking, alcohol use, or illicit drug use. He had no history of tattoos, acupuncture, or prior blood transfusion.

**Figure 1:**
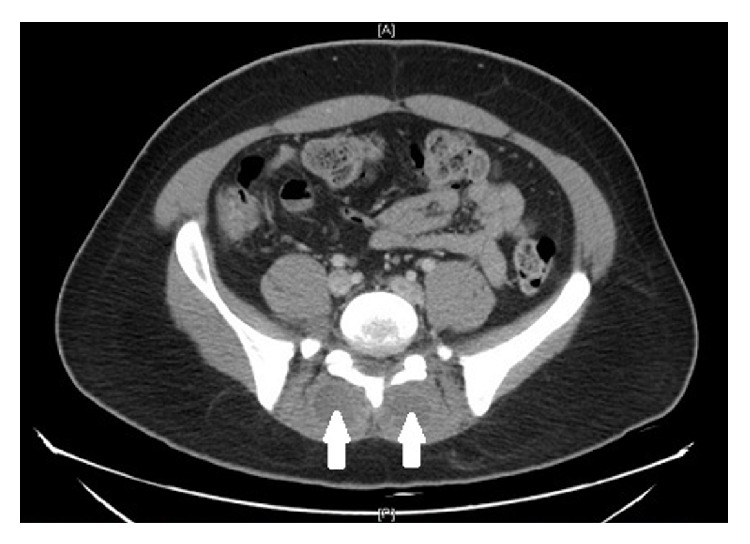
CT of the abdomen with two arrows showing the areas of low-density within the posterior paraspinal muscles.

**Figure 2:**
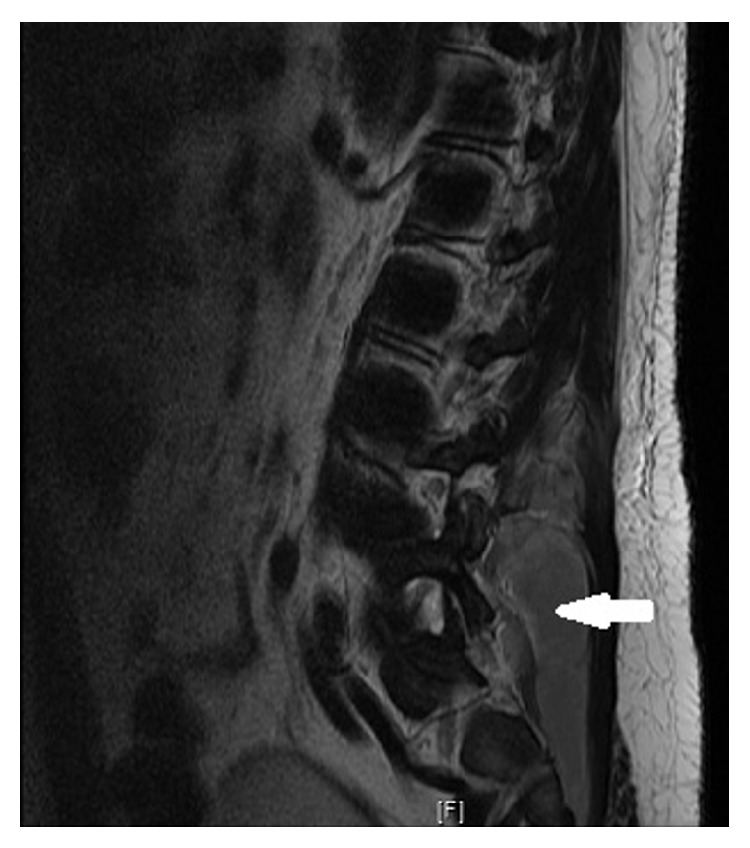
Sagittal view of T2 diffusion MRI lumbar spine showing heterogeneous lesions (arrow) involving the paraspinal muscles with a ring enhancement consistent with abscess.

**Figure 3:**
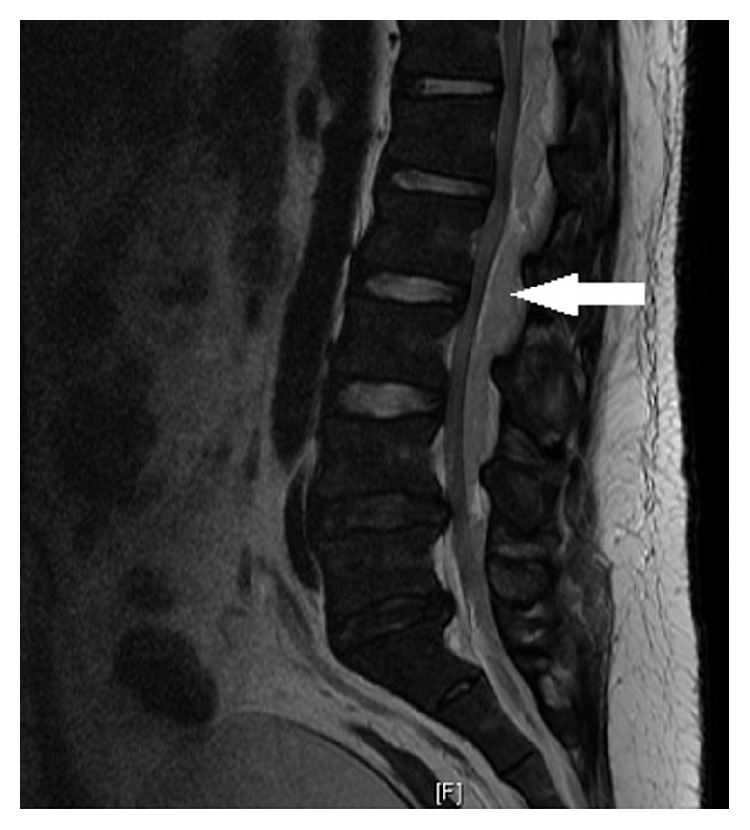
Sagittal view of T2 diffusion MRI lumbar spine showing extensive epidural abscess (arrow) extending from the upper dorsal region all the way to the distal conus medullaris, displacing the thecal sac.

Upon initial admission, his vital signs were temperature 99.8 F, heart rate 83 bpm, respiratory rate 19/minute, blood pressure 136/69, and oxygen saturation 97% on room air. Pertinent positive on physical exam was tenderness in umbilical region and mild to moderate discomfort in the right and left flank on deep palpation. He also had two small pustular lesions on his neck and chin which he attributed to his chronic acne. He did not have costovertebral, spinal, and paraspinal tenderness, knocking pain in the flanks, organomegaly, or Murphy's sign. He had no limited range of motion in the lumbar spine. The straight leg test did not elicit pain in the lower back. There was no focal finding on a complete neurological examination. His gait was completely intact. Rectal tone was also intact with no fecal impaction. The initial laboratory results showed a white blood cell count of 27 k/uL, sedimentation rate of 72 mm/hr, C-reactive protein (CRP) of 35.5 mg/dL, lipase of 87 U/L, albumin of 2.9 g/dL, and normal liver function. Initial blood and urine cultures were drawn. Computerized tomography (CT) scan of the abdomen showed a small 2 mm nonobstructing calculus within the right kidney, mild anterior wedging of L1 and T12 consistent with mild compressions, posterior bulging annulus at L4-L5 with apparent moderate canal narrowing, and areas of low-density within the posterior paraspinal muscles (Figure 1). Because of the concern for spinal pathology on a CT scan, magnetic resonance imaging (MRI) lumbar was done. It showed extensive extradural collection involving the dorsal lumbar spine with probably extensive epidural abscess extending from the upper dorsal region all the way to the distal conus medullaris, displacing the thecal sac anteriorly (Figures 2 and 3). There were also heterogeneous lesions involving the paraspinal muscles bilaterally with a ring enhancement consistent with abscesses.

Patient was started on empiric antibiotic with IV vancomycin and piperacillin-tazobactam. With patient's consent, he was taken into the operative room for emergency neurosurgical intervention. He had a T1-S2 decompressive laminectomy with drainage of epidural abscess and bilateral drainage of lumbosacral paraspinal muscle abscesses. The initial blood cultures and surgical cultures grew methicillin sensitive* Staphylococcus aureus*. Transesophageal echocardiogram did not show any valvular vegetation. Antibiotics were switched to nafcillin. The patient started ambulating on postoperative day 2. He was subsequently discharged home with physical therapy and IV nafcillin for total of six weeks. Repeat MRI at six weeks showed resolution of both the paraspinal and epidural abscesses.

## 3. Discussion

Paraspinal and epidural abscesses are rare conditions that can often be misdiagnosed unless physicians have high index of suspicion. Generally, well accepted staging systems used for outlining the progression of symptoms for epidural abscesses are (1) spinal pain at affected level, (2) radicular pain, (3) sensory motor deficit below the lesion with neurogenic bladder or bowel, and (4) paralysis and death [9]. In rare occasions, the radicular symptoms such as chest or abdominal pain can be the only presenting symptoms. The mechanism of these radicular pains is through dermatomal distribution of the affected spinal area. It is often challenging to diagnose in the early stages of the disease process unless clinicians have high index of suspicion.

The incidence of abdominal pain associated with spinal epidural abscess is rare, with only a few case reported. Hagan and Adjogatse reported a 47-year-old male who underwent laparotomy for an acute abdomen and was subsequently found to have an epidural abscess [2]. Prasad and De Vere also reported a similar case in a pediatric patient [8]. Four other cases have been reported on spinal epidural abscess presenting with abdominal pain as the chief complaint [5–7, 10]. However, never in history has there been a case report on paraspinal abscess with extensive epidural abscess presented with vague abdominal pain.

In our case, the patient did not have any risk factors and his presenting symptoms were very vague which led us to initially investigate for intra-abdominal pathologies. The additional complaints of urinary retention and constipation were “red flags” in which we paid valuable attention in our evaluation. We arrived to our final diagnosis after carefully evaluating the patient's septic profile with severe leukocytosis, elevated CRP, and a suspicious CT of abdomen finding and finally deciding to proceed with MRI imaging in a sequential manner. We speculated that our patient had transient bacteremia from pustular acne and bacteria seeded into the paraspinal muscle due to mechanical back injury. However, due to the lack of history provided by the patient, we cannot specifically pinpoint the source of bacteremia and abscess formation.

Even paraspinal abscess and large epidural abscess can be present with a subtle complaint as in the case of our patient. This case highlights the importance of carefully evaluating vague abdominal pain in the absence of abdominal pathology as this could mimic a paraspinal and epidural abscess. Diagnosis should necessitate a high clinical index of suspicion. Early diagnosis is the key to an optimal outcome with regard to paraspinal and epidural abscesses.
